# Interpretation of Social Interactions: Functional Imaging of Cognitive-Semiotic Categories During Naturalistic Viewing

**DOI:** 10.3389/fnhum.2018.00296

**Published:** 2018-08-14

**Authors:** Dhana Wolf, Irene Mittelberg, Linn-Marlen Rekittke, Saurabh Bhavsar, Mikhail Zvyagintsev, Annina Haeck, Fengyu Cong, Martin Klasen, Klaus Mathiak

**Affiliations:** ^1^Department of Psychiatry, Psychotherapy and Psychosomatics, Medical Faculty, RWTH Aachen University, Aachen, Germany; ^2^Natural Media Lab, Human Technology Centre (HumTec), RWTH Aachen University, Aachen, Germany; ^3^Center for Sign Language and Gesture (SignGes), RWTH Aachen University, Aachen, Germany; ^4^Brain Imaging Facility, Interdisciplinary Centre for Clinical Studies (IZKF), Medical Faculty, RWTH Aachen University, Aachen, Germany; ^5^Department of Biomedical Engineering, Faculty of Electronic Information and Electrical Engineering, Dalian University of Technology, Dalian, China; ^6^JARA-Translational Brain Medicine, Aachen, Germany

**Keywords:** social cognitive neuroscience, natural film viewing, functional imaging, semiotics, social interaction

## Abstract

Social interactions arise from patterns of communicative signs, whose perception and interpretation require a multitude of cognitive functions. The semiotic framework of Peirce’s Universal Categories (UCs) laid ground for a novel cognitive-semiotic typology of social interactions. During functional magnetic resonance imaging (fMRI), 16 volunteers watched a movie narrative encompassing verbal and non-verbal social interactions. Three types of non-verbal interactions were coded (“unresolved,” “non-habitual,” and “habitual”) based on a typology reflecting Peirce’s UCs. As expected, the auditory cortex responded to verbal interactions, but non-verbal interactions modulated temporal areas as well. Conceivably, when speech was lacking, ambiguous visual information (unresolved interactions) primed auditory processing in contrast to learned behavioral patterns (habitual interactions). The latter recruited a parahippocampal-occipital network supporting conceptual processing and associative memory retrieval. Requesting semiotic contextualization, non-habitual interactions activated visuo-spatial and contextual rule-learning areas such as the temporo-parietal junction and right lateral prefrontal cortex. In summary, the cognitive-semiotic typology reflected distinct sensory and association networks underlying the interpretation of observed non-verbal social interactions.

## Introduction

^1^During social interactions, a multitude of auditory and visual cues interact to convey meaning. These cues range from spoken words and manual gestures to facial expressions, eye gaze, body orientation, and body movements (Lotze et al., 2006; Schilbach et al., 2006; Becchio et al., 2012; Saggar et al., 2014). Interpreting social interactions requires the interaction of various cognitive functions; among these are social attention mechanisms, mentalizing, language comprehension, and the recognition of faces, communicative gestures, goal-directed movements, and emotions (Ochsner and Lieberman, 2001; Adolphs, 2009; Hari and Kujala, 2009). In a laboratory setting, naturalistic stimuli such as movies or film sequences capture this complexity because they provide a means to represent the interacting information dynamically and within context (Spiers and Maguire, 2007; Hasson and Honey, 2012; Willems, 2015). With regard to social interactions in complex scenes, validated comprehensive typologies are lacking and their neural processing is unclear.

During watching movies or film sequences, different neural networks were found involved depending on the study protocol and stimulus material. The most consistently reported neural correlates of social-cognitive functions encompass superior temporal gyrus (STG), temporo-parietal junction (TPJ), medial prefrontal cortex (PFC), fusiform gyrus, and precuneus (Iacoboni et al., 2004; Wolf et al., 2010; Lahnakoski et al., 2012; Wagner et al., 2016). In particular, the posterior STG and TPJ of the right hemisphere serve as key regions during the processing of real-life social interactions and joint attention (Redcay et al., 2010; for a meta-analysis see Krall et al., 2015). Both regions may contribute to the analysis of social relations in movie clips (Iacoboni et al., 2004). Specific social signals in movies (e.g., faces, movement, social interactions, or speech) may even partially segregate four networks (Lahnakoski et al., 2012): Scenes depicting social interactions engaged two of those networks, namely a temporal-amygdala network and a prefrontal-insula network. The introduction of an explicit mentalizing task modulated neuronal recruitment patterns (Wolf et al., 2010).; rating an agent’s intention activated three independent neural networks, i.e., for face processing and recognition, language comprehension, as well as self-referential mental activity. Taken together, the significance of several cortical and subcortical structures for the processing of social interaction has become evident, but their specific contribution to the interpretation of interaction events, especially during naturalistic stimulation, remains to be determined.

Although social interactions comprise several interwoven, multimodal cues, a behavioral pattern, as a whole, acts as a communicative sign and can thus be described using sign theory (semiotics). Semiotic categories describe cognitive categories, among other related phenomena (Holenstein, 2008). Thus, semiotic models are amenable to cognitive-behavioral and neuroscientific testing (Paolucci, 2011; Galantucci et al., 2012; Zlatev, 2012). Among one of the most influential contributions to the field of semiotics are the UCs introduced by Charles Sanders Peirce (Peirce, 1955, 1960). Peirce’s theory has been considered an appropriate semiotic framework for the study of cognitive processes (Daddesio, 1994; Stjernfelt, 2007; Fusaroli and Paolucci, 2011; Sonesson, 2014) and has been applied to theoretical and empirical investigations in the fields of cognitive semiotics, linguistics, media science, and neurosciences. This semiotic theory is well suited for neuroimaging studies of multimodal communication because it emphasizes the perspective of the interpreting mind (Peirce, 1955), represented by the observers brain activity. This framework may describe the relation between perceived signs (e.g., the interactions shown in movie clips) and the *interpretant* (Peirce, 1955), i.e., the resulting cognitive representation in the participant’s mind and subsequent brain activity during a functional magnetic resonance imaging (fMRI) experiment.

**FIGURE 1:**
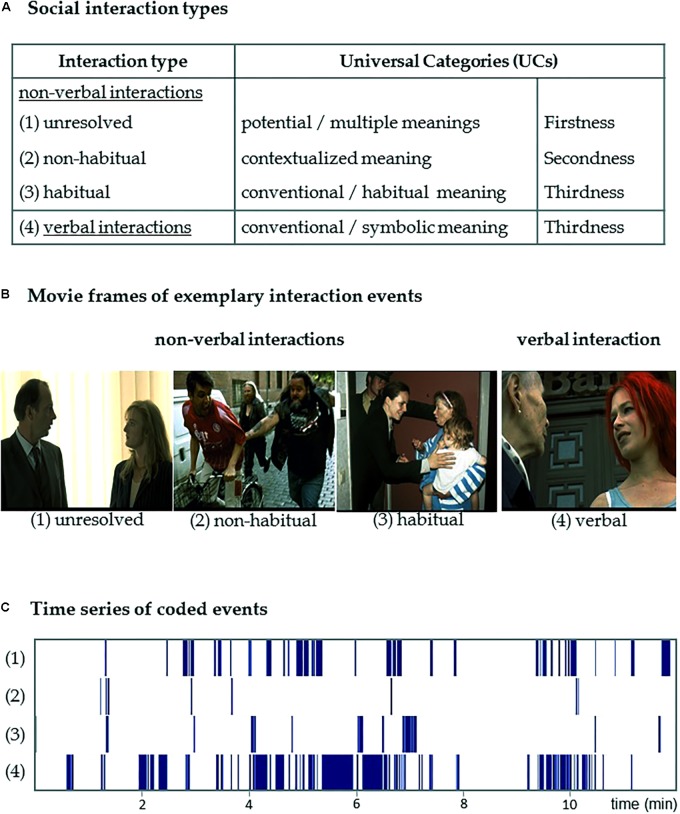
Coded interaction types. Social interaction events occurring in the movie excerpt were annotated. **(A)** Peirce’s semiotic framework of the Universal Categories inspired the differentiation of three cognitive processes during the interpretation of non-verbal and one during verbal social interactions. (1) Unresolved non-verbal interactions are ambiguous and the social situation is not yet resolved (reflecting the Firstness category of potential, not-yet-resolved meaning); (2) “non-habitual interactions” counteract learned behavioral patterns and are disambiguated by the local context (reflecting the Secondness category of contextualized meaning); (3) “habitual interactions” rely on learned behavioral patterns and social conventions (reflecting the Thirdness category of conventional and habitual meaning); and, finally (4) words rely on the conventional codes of the language and thus verbal interaction reflect always the Thirdness category. **(B)** The movie frames depict exemplary events for each of the coded types of social interactions: (1) unresolved: two people wordlessly stare at each other; (2) non-habitual: a cyclist is grabbed by a passerby; (3) habitual: a carried child is touched in greeting; and (4) verbal: two persons engage in a conversation. **(C)** Visualization of the time series of coded events in the 12-min movie excerpt. Copyrights of screen shots: Tykwer (1998).

With regard to the representation, transmission, and interpretation of signs, Peirce’s UCs distinguish between three levels: “Firstness” pertains to potential, not-yet-resolved meaning; “Secondness” encapsulates the specific, contextualized meaning of a sign, particularly of non-habitual and non-conventional signs; and “Thirdness” involves entrenched habits, patterns, and rules (Peirce, 1960). While semiotic categories typically interact to various degrees in a given sign process, one of them can be expected to predominate and, thus, determine the sign’s main function, as well as the way it is perceived and interpreted (Peirce, 1955). We categorized non-verbal social interactions occurring within a film sequence into three types (**Figure 1**): (1) “unresolved” interactions are ambiguous in the respective situation and their outcomes are not yet determined (emphasizing Firstness); (2) “non-habitual” interactions counteract learned behavioral patterns and are disambiguated by the local context (emphasizing Secondness); and (3) “habitual interactions” include implicitly or explicitly learned behavioral patterns, which conform to social conventions (emphasizing Thirdness). (4) Verbal interactions largely rely on the conventional codes of a given language and culture and are subsumed in a forth category (high degree of Thirdness; see **Figure 1**). This operationalization aims at the description of the sign-interpretant relation with respect to the UCs. We yielded three processing modes for the interpretation of non-verbal interactions and one for all verbal interactions, because spoken language is highly conventionalized and dominated by the Thirdness category.

Peirce’s UCs were chosen as the theoretical background to study social interactions because they have inspired various theoretical models that have been widely used to describe and interpret communicative actions and signs. However, experimental evidence is still scarce for Peirce’s basic constructs. Peirce aimed at a general semiotic theory that accounts for all kinds of sign processes in all kinds of modalities, including those occurring in nature and scientific inquiry; hence, his model of sign processes goes beyond communication *per se* (Jensen, 1995; Nöth, 2001). The UCs have inspired theoretical models developed to characterize and interpret manual gestures (e.g., McNeill, 1992, 2005; Fricke, 2007; Mittelberg, 2008, 2013, 2018; Mittelberg and Waugh, 2014), onomatopoeia in language (Jakobson, 1966; Guynes, 2013), and images (Sonesson, 2005; Jappy, 2011), and further, describe narrative comprehension in both spoken and written stories (Lee, 2012), film sequences (Deleuze, 1986, 1989; Sykes, 2009) and comics (Magnussen, 2000; Cohn, 2007; see also Bateman et al., 2017 on multimodality). Qualitative analyses utilized aspects of Peirce’s UCs for the investigation of mental imagery, human gestures, language evolution, and developmental aspects of communication and culture (for a review, see Zlatev, 2012). However, despite many theoretical and qualitative approaches, empirical investigations are still rare. Best known, the sign-object relation is one aspect of the UCs, and has founded a prominent theoretical framework for empirical analyses of manual gestures (as “iconic” (UC1), “deictic” (UC2), and “emblematic” (UC3) gestures; McNeill, 1992) in behavioral and neuroimaging studies on gesture perception and comprehension (for reviews, see Özyürek, 2014; Wagner et al., 2014; Yang et al., 2015). The sign-interpretant relation of the UCs has scarcely been used as a construct for neuroimaging studies. We investigated conventionality in co-speech gestures with the sign-interpretant relation of thirdness (UC3, Wolf et al., 2018). Perceiving a gesture as conventional increased intersubject covariance (ISC) in left inferior frontal gyrus (IFG) and posterior STG. The present study employed aspects of the UCs to create stimulus categories in a neurocognitive experiment. Social interactions were labeled with semiotic categories derived from the UCs and their neural response patterns were analyzed.

Assuming that semiotic categories influence the cognitive and neural processing of the interpretant, the semiotic characterization of social interactions differentiates neural processes during the observation of social interactions. Functional MRI recorded neural responses to social interactions portrayed during a 20-min movie narrative. The three types of non-verbal interactions (unresolved, non-habitual, and habitual) and any verbal interactions were coded. First, we aimed to confirm that this method yields neural correlates of social interactions encompassing the relevant sensory, language, visual, and social cognitive networks. In specific, we hypothesized that brain areas supporting the interpretation of verbal interactions lean more toward auditory processes whereas non-verbal interactions elicit stronger visuo-spatial processing. Second, the recruitment of these brain areas during the observation of non-verbal interactions was expected to depend on the predominant semiotic category (visible by the extracted beta values). Third, we explored neural patterns contributing to the encoding of Peirce’s semiotic categories in a whole-brain analysis.

## Materials and Methods

### Study Participants

Sixteen right-handed native German speakers (seven women, age 26.1 ± 3.8, range 22–34 years) participated in the present study. Participants had normal or corrected-to-normal vision, normal hearing, and no history of psychiatric, neurological, or mental disorders. The study protocol was approved by the local Ethics Committee and the experiment was designed and conducted in accordance with the Declaration of Helsinki. All participants gave written informed consent and received financial reimbursement.

### Stimulation

Participants were presented with a film sequence showing a 20-min excerpt from the German movie “Lola rennt” (Engl.: “Run Lola Run”; 10:20–30:20 min; X-Filme Creative Pool, Germany, 1998). The movie excerpt was chosen because it comprises a self-contained narrative with a fast-paced story line within a reasonable time frame (20-min). Video was delivered by a projector system with reflecting mirrors (Psychology Software Tools, Sharpsburg, PA, United States), and audio was delivered by earplugs (Nordic Neurolab Bergen, Norway). The sound was individually adjusted to a comfortable hearing level. Stimulus delivery and timing was controlled using the stimulation software Presentation (Neurobehavioral Systems Inc., United States). Before the film sequence started, a fixation cross was presented for 25 s. The participants were asked to watch attentively.

### Coding of Social Interactions in the Movie

A film sequence is a naturalistic stimulus in which a multitude of features interact. In order to model the specific appearance of social interactions we used an established content-coding approach for model-based analysis (Mathiak and Weber, 2006; Weber et al., 2009; Mathiak et al., 2011, 2013; Klasen et al., 2012b). We annotated the onsets and duration of social interaction events in the movie excerpt on a frame-by-frame basis with 67 ms accuracy, corresponding to a 15 Hz frame rate. The content-coding system distinguished three types of non-verbal social interactions: (1) unresolved, (2) non-habitual, and (3) habitual; as well as four verbal interactions (for an overview, see **Figure 1**). For each event, exactly one of the interaction types was annotated. Verbal interactions were coded whenever speech was involved in the interaction event. The duration of a verbal interaction event corresponded to the duration of the utterance. Pauses up to two seconds between words or utterances were coded as continuous verbal interaction. Pauses lasting longer than two seconds were coded as a non-verbal interaction. Non-verbal interactions could immediately precede or follow a verbal interaction (e.g., an “unresolved interaction” is followed by a “verbal interaction”). Furthermore, non-verbal interaction types may immediately follow each other (e.g., an “unresolved interaction” is followed by a “habitual interaction”). The complete movie excerpt was annotated twice by two independent coders. Inter-coder reliability for the differentiation between interaction types was determined with Krippendorff’s alpha (.62). In cases when the coders disagreed, a supervisory decision was taken by a third coder (D.W.). In total, 170 interaction events were annotated yielding a total duration of 476.5 s (average duration: 2.8 ± 5.8 s, mean ± SD). Of those interaction events, 65 were coded as “unresolved” (total duration: 132.6 s; average: 1.7 ± 2.5 s), 19 events were coded as “non-habitual” (total duration: 10.0 s; average: 0.5 ± 0.6 s), 22 events were coded as “habitual” (total duration: 42.2 s; average: 1.9 ± 2.5 s), and 64 events were coded as “verbal” (total duration: 291.7 s; average: 4.6 ± 8.8 s).

### MR Data Acquisition

Functional MRI was conducted using a 3 Tesla Siemens Scanner (Magnetom Trio, Siemens Medical Systems, Erlangen, Germany) and a 32-channel phased-array receive-only head coil. Echo planar imaging (EPI) collected functional images sensitive to the blood-oxygenation-level-dependent (BOLD) contrast. The applied EPI sequence acquired multiple echoes after a single excitation pulse. Subsequently, the obtained images were weighted and combined. This procedure increases signal-to-noise ratio (Bhavsar et al., 2014), which may be particularly beneficial for investigations that utilize naturalistic stimuli. With the following parameters, 487 volumes were acquired: 24 slices; echo time (TE) = 17.0, 45.9, and 74.9 ms; repetition time (TR) = 2540 ms; flip angle (FA) = 90°; slice thickness = 3.5 mm; slice gap = 0.5 mm; matrix size = 64 × 64, field of view (FOV) = 224 mm^2^ × 224 mm^2^; voxel size = 3.5 mm^3^ × 3.5 mm^3^ × 3.5 mm^3^, and bandwidth = 2232 Hz/pixel.

### Data Analysis

Functional MRI data analysis was conducted using the software Statistical Parametric Mapping (SPM 8, Wellcome Department of Imaging Neuroscience UCL, London, United Kingdom). The first five volumes of each session were discarded to reduce T1 saturation effects. Images were spatially realigned, normalized to the stereotactic MNI space (Montreal Neurological Institute; Evans et al., 1993) and resampled to 2 mm × 2 mm×2 mm voxels. Spatial smoothing with a full-width-at-half-maximum (FWHM) Gaussian kernel of 12 mm was applied to the normalized data. The single-echo images were combined after normalization based on the optimized-CNR approach, i.e., each image voxel was weighted with a function of the estimated signal-to-noise ratio and the expected BOLD sensitivity (Mathiak et al., 2004; Poser et al., 2006; see Bhavsar et al., 2014 for detailed methodological descriptions).

The onset and duration of each coded event entered a general linear model (GLM) with one predictor of interest for each interaction type (unresolved, non-habitual, habitual, and verbal). Using the canonical response function, a model for the BOLD responses to the events was constructed. In the first-level statistical analysis, the contrast of the four stimuli types against the baseline (the ongoing movie) was determined. The four contrast maps of each subject entered the repeated-measures ANOVA for second-level analysis. An *F*-test determined effects of the four interaction types against baseline. *Post hoc* *t*-tests compared the verbal with the non-verbal interactions. A second *F*-test explored the activation patterns differentiating the semiotic categories of observed non-verbal interactions. *Post hoc* *t*-tests compared non-verbal interaction types in a pairwise fashion. Each map in the second-level group analyses was corrected with a voxelwise family-wise error (FWE; Friston et al., 1994) of *p* < 0.05. All remaining clusters survived a clusterwise *p*_FWE_ < 0.05 thresholding.

Region-of-interest (ROI) analyses investigated differences between the three non-verbal interaction types. Therefore, the selected ROIs differentiated the verbal from the non-verbal interactions and vice versa. For the verbal condition, peak locations were obtained from the contrast “verbal > non-verbal.” For the non-verbal conditions, the inversed contrast “non-verbal > verbal” was considered; extended clusters were segregated into meaningful anatomical structures according to the AAL atlas (Tzourio-Mazoyer et al., 2002): fusiform gyrus (bilateral), calcarine gyrus (bilateral), right IFG pars triangularis (IFG PTr), and right middle frontal gyrus (MFG). Due to the orthogonality of the SPM second-level design matrix, we assumed orthogonality of the estimate for the “verbal versus non-verbal” contrast with the one for the contrasts between the non-verbal subtypes; therefore the data were explored with standard univariate ANOVAs. Significant differences across the non-verbal interaction types were determined with one-factor ANOVAs and corrected for multiple comparisons according to Bonferroni-Holm. Statistics were performed with IBM SPSS (Statistics for Windows, Version 20.0, Armonk, NY, United States).

In a supplementary analysis the validity of the obtained neural functioning for the processing of verbal information and social interaction was confirmed with anatomical masks from Neurosynth^2^; this toolbox provides neuroanatomical information based on meta-analysis of fMRI studies. The images for the terms “verbal” (615 studies) and “social interaction” (94 studies) were merged and served for small-volume correction of the contrasts “verbal > non-verbal” and “non-verbal > verbal” as well as the individual *t*-contrasts comparing the non-verbal interaction types. Segregation of anatomical structures and the extraction of ROI data were performed as described above. Significant differences in peak-voxel activation were explored with univariate ANOVAs.

## Results

### Neural Responses to the Observation of Social Interactions

**FIGURE 2:**
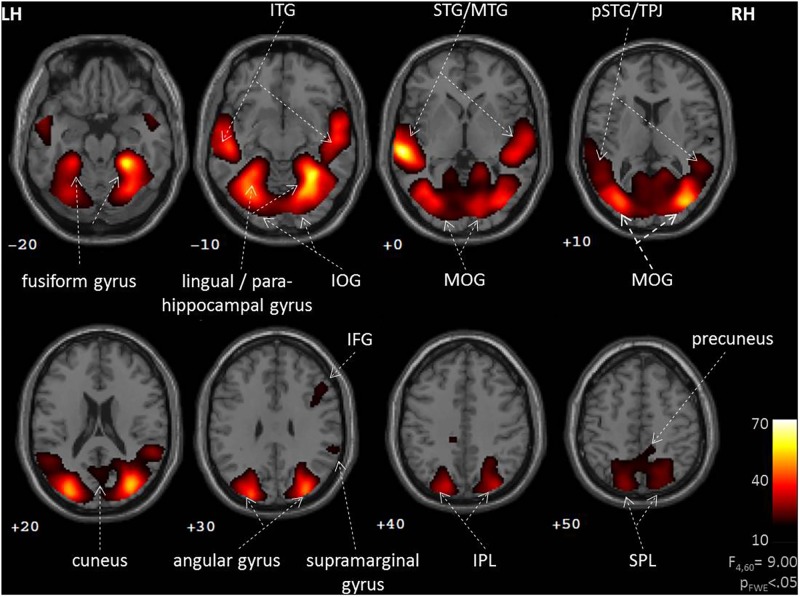
Neural correlates of social interactions. Social interaction events in a movie narrative were coded as four types (unresolved, non-habitual, habitual, and verbal). An *F*-test across the four predictors revealed widespread brain activation of auditory and visual networks. Such pattern is commonly observed during the perception of naturalistic social stimuli, suggesting an important role for the observation of social interaction events. LH, left hemisphere; RH, right hemisphere; IFG, inferior frontal gyrus; IOG, inferior occipital gyrus; IPL, inferior parietal lobe; ITG, inferior temporal gyrus; MOG, middle occipital gyrus; MTG, middle temporal gyrus; (p)STG, (posterior) superior temporal gyrus; SPL, superior parietal lobe; TPJ, temporo-parietal junction; *z*-coordinates are indicated beneath each slice.

**FIGURE 3:**
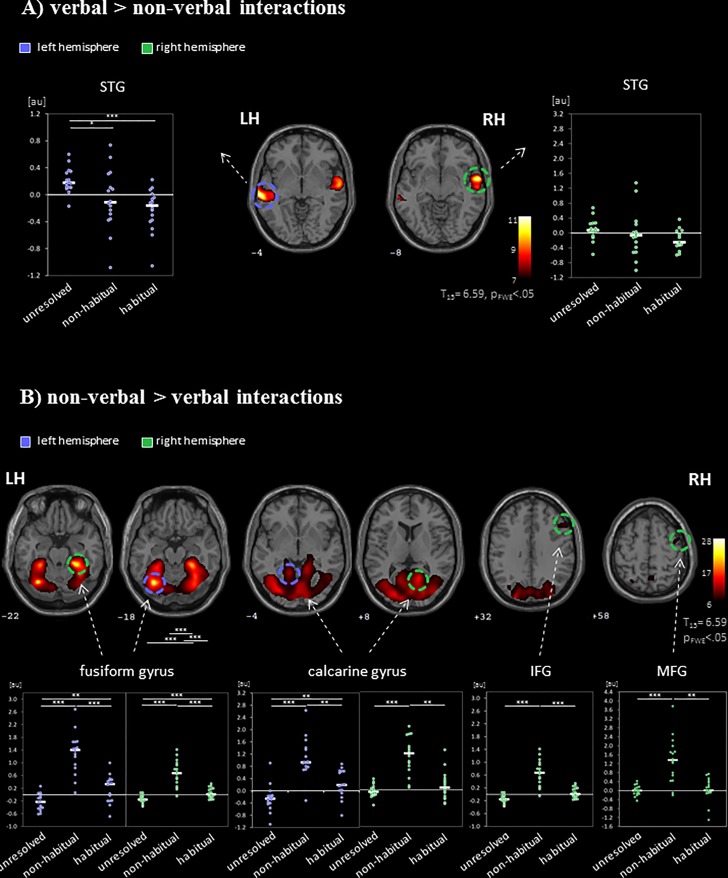
Neural networks for the processing of verbal and non-verbal interactions. The contrasts between **(A)** verbal (“verbal > non-verbal”) and **(B)** non-verbal (“non-verbal > verbal”) interactions separated the networks for visual-based and sound-based information processing. ROI analyses of regional peak voxels revealed specific contributions for the non-verbal interaction types (“unresolved,” “non-habitual,” and “habitual”). Auditory regions were recruited during the observations of unresolved interactions but suppressed during habitual interactions. Non-habitual interactions yielded the involvement of visual and prefrontal areas. LH, left hemisphere; RH, right hemisphere; IFG, inferior frontal gyrus; MFG, middle frontal gyrus; STG, superior temporal gyrus; ^∗^*p* < 0.05; ^∗∗^*p* < 0.01; ^∗∗∗^*p* < 0.001; *z*-coordinates are indicated beneath each slice.

During social interactions, several brain regions exhibited activity increases, including primary auditory and visual areas as well as higher order visual pathways (**Figure 2** and **Table 1**). Previous studies reported medial PFC activations (e.g., Iacoboni et al., 2004), which emerged in our data only after lowering the threshold to *p* < 0.001 uncorrected. Differences between verbal and non-verbal interactions were investigated with *t*-tests contrasting responses to verbal interactions against the average response to the three non-verbal interaction types. The contrast “verbal > non-verbal” yielded strong bilateral activation in auditory processing areas (STG extending into middle temporal gyrus (MTG); **Figure 3A** and **Table 2**). The reversed contrast “non-verbal > verbal” yielded widespread brain regions, in particular, the visual pathway emerged encompassing occipital, inferior temporal, and superior parietal cortices as well as right prefrontal areas (**Figure 3B** and **Table 2**).

**Table 1:** Cluster table for *F*-tests.

Peak voxel location	Cluster size [voxel]	Peak *F*-value	Peak voxel		
			*x*	*y*	*z*
***F*-test across interaction types (**Figure 2**)**					
Superior temporal gyrus L^∗^	39,566	74.17	−62	−26	−2
Fusiform gyrus R^∗^		62.72	28	−40	−16
Inferior frontal gyrus PTr R	409	11.98	48	22	30
Middle frontal gyrus R		4.87	56	24	36
Precentral gyrus R		4.36	38	0	42
Middle cingulate cortex L	92	11.41	−12	−32	40
Middle frontal gyrus R	13	9.44	46	2	60
***F*-test across non-verbal interaction types (**Figure 4**)**					
Fusiform gyrus R^∗^	21,862	65.49	30	−62	−14
Fusiform gyrus R^∗^		60.90	32	−50	−12
Inferior occipital gyrus L^∗^		57.31	−36	−66	−8
Inferior frontal gyrus PTr R	957	21.34	50	22	30
Middle frontal gyrus R		17.30	46	2	60
Precentral gyrus R		16.85	36	0	40
Superior parietal lobe R	34	15.02	28	−56	48

*Clusters with a minimum cluster size of 10 voxels and located within the brain mask are reported. *T*- or F-values are reported at p_FWE_ < 0.05; peak voxel coordinates are given in MNI-space. ^∗^Cluster extends to other hemisphere. PTr, pars triangularis.*

**Table 2:** Cluster table for comparison between verbal and non-verbal interactions.

Peak voxel location	cluster size	peak *t*-value	peak voxel		
	[voxel]		*x*	*y*	*z*
**Verbal > non-verbal interaction types** (**Figure 3A**)					
Superior temporal gyrus L	946	11.60	−62	−26	−2
Superior temporal gyrus R	767	10.61	62	0	−8
**Non-verbal > verbal interaction types** (**Figure 3B**)					
Cerebellum L^∗^	235190	28.16	−32	−64	−20
Fusiform gyrus R^∗^		23.91	28	−38	−22
Fusiform gyrus L^∗^		18.45	−28	−38	−22
Inferior frontal gyrus PTr R	47	7.43	56	26	34
Middle frontal gyrus R	16	6.79	44	0	58
Precuneus R	13	6.74	2	−60	54

*Clusters with a minimum cluster size of 10 voxels and located within the brain mask are reported. T- or F-values are reported at p_FWE_ < 0.05; peak voxel coordinates are given in MNI-space. ^∗^Cluster extends to left hemisphere. PTr, pars triangularis.*

To differentiate the effect of the three non-verbal interaction types, a ROI analysis was performed in the peak-voxels of the contrasts between verbal and non-verbal interactions. Verbal interactions yielded higher responses in bilateral auditory cortices than non-verbal ones [left: MNI (*x,y,z*) = −62, −26, −2 (STG); right: 62, 0, −8 (STG); **Figure 3A**]. In the left hemispheric ROI only, a modulation with respect to the three non-verbal interaction types emerged (left: *F*_2,45_ = 7.00, *p* = 0.002; right: *F*_2,45_ = 1.78, *p* = 0.18, n.s.). The activation differences were characterized by a gradient from unresolved to non-habitual to habitual interactions (see inserts in **Figure 3A**): In *post hoc* *t*-tests, unresolved interactions yielded higher activity as compared to habitual interactions (*t*_15_ = 5.28, *p* < 0.001) and to non-habitual interactions (*t*_15_ = 2.60, *p* = 0.02; habitual versus non-habitual: *t*_15_ = 0.98, *p* = 0.344, n.s.). For the non-verbal contrast, six ROIs emerged: left fusiform gyrus (−32, −64, and −18), right fusiform gyrus (28, −38, and −22), left calcarine gyrus (2, −80, and −4), right calcarine gyrus (16, −56, and 8), right IFG PTr (54, 24, and 32), and right MFG (44, 0, and 58). In each ROI a significant difference between the non-verbal conditions emerged (all *p* < 0.001; **Figure 3B**). In the *post hoc t*-test, non-habitual interactions yielded larger activation than the two other types (all *t*_15_ > 3.80, *p* < 0.003; see inserts in **Figure 3B**). After applying the Neurosynth-based mask, two ROIs emerged for verbal interactions over nonverbal interactions (peak voxels: left STG: −62, −26, and −2; right STG: 62, −2, and −6) and five ROIs for non-verbal interactions (peak voxels: left fusiform gyrus: −32, −62, and −18; right fusiform gyrus: 30, −62, and −16; left calcarine gyrus: −10, −90, and 4; right calcarine gyrus: 14, −68, and 8; right IFG PTr: 54, 24, and 32). The Neurosynth-mask did not cover the cluster detected in the right MFG. In each of the remaining ROIs, the significant differences between the non-verbal conditions as well as the larger activation for non-habitual interactions were confirmed (all *p* < 0.001; **Supplementary Figure S1**). Thus, the functional responses obtained during watching naturalistic stimulation were in accordance with meta-analytical maps for verbal stimulation and social interactions. The widespread activation of cortical regions during the processing of non-verbal interactions was grounded in the processing of non-habitual interactions.

### Brain Regions Underlying UCs in Non-verbal Interactions

**FIGURE 4:**
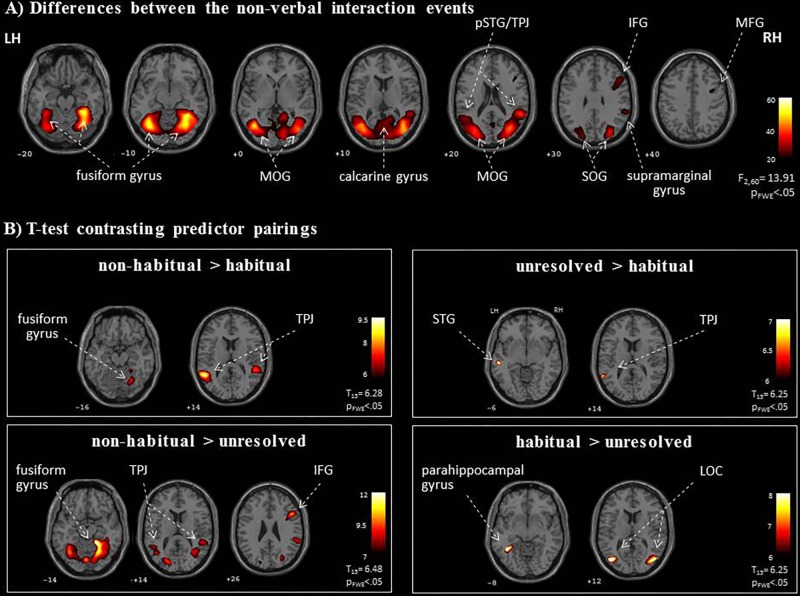
Neural networks for the processing of non-verbal interactions. **(A)** The specific *F*-test differentiated processing networks for the semiotic categories (“unresolved,” “non-habitual,” and “habitual”). **(B)** *Post hoc t*-maps determined specific effects in four directed contrasts. As an extension to the ROI analysis, the TPJ may be relevant for the interpretation of unresolved or non-habitual interactions, whereas the hippocampus and the LOC responded most strongly to habitual interactions. LH, left hemisphere; RH, right hemisphere; IFG, inferior frontal gyrus; LOC, lateral occipital complex; pSTG, posterior superior temporal gyrus; MFG, middle frontal gyrus; MOG, middle occipital gyrus; SOG, superior occipital gyrus; TPJ, temporo-parietal junction; *z*-coordinates are indicated beneath each slice.

We further explored brain regions that engaged differentially in the encoding of the semiotic categories of the observed non-verbal social interactions. Thereto, we conducted mappings of the differences between the three conditions with an *F*-test and *post hoc t*-tests. The *F*-test yielded an extended pattern of brain regions with the highest activations in the fusiform gyrus and middle occipital gyrus (MOG; see **Figure 4A** and **Table 1**), similar to those obtained for the contrast between non-verbal and verbal interactions (compare with **Figure 3B**). Of the six directed comparisons in the *post hoc t*-tests, four yielded significant clusters at a threshold according to a *p*_FWE_ < 0.05 (**Figure 4B** and **Table 3**). First, specific contributions of both other types – anticipation and non-habituality – emerged when contrasted to habitual interactions: Neural responses were increased in the left STG and TPJ during unresolved interactions (right upper panel in **Figure 4B**) and in the right fusiform gyrus and bilateral TPJ during non-habitual interactions (left upper panel in **Figure 4B**). Using the unresolved condition as baseline, habitual interactions yielded higher activation in the left parahippocampal gyrus and bilateral MOG (right lower panel in **Figure 4B**), and non-habitual interactions elicited higher activation in the right IFG as well as bilateral fusiform gyrus, TPJ, and MOG (left lower panel in **Figure 4B**). The contrasts “unresolved > non-habitual” and “habitual > non-habitual” did not yield differences at the same threshold. After correction of the contrasts with the Neurosynth-based mask, each of the detected clusters fell – at least partly – into the brain mask (**Supplementary Figure S2** and **Supplementary Table S1**). Involvement of the reported brain regions in processing of verbal information and social interaction was thereby validated based on this meta-analytical approach.

**Table 3:** Cluster table for comparison between non-verbal interaction types (**Figure 4**).

Peak voxel location	Cluster size [voxel]	Peak *T*-value	Peak voxel		
			*x*	*y*	*z*
**Unresolved > habitual interactions**					
Superior temporal gyrus L	55	7.00	−56	−26	−6
Temporo-parietal junction L	19	6.71	−58	−52	14
**Habitual > unresolved interactions**					
Parahippocampal gyrus L	127	8.96	−40	−50	−8
Middle occipital gyrus L		6.63	−38	−62	−2
Middle occipital gyrus L	366	8.90	−36	−74	18
Middle occipital gyrus R	455	8.29	48	−74	10
**Non-habitual > habitual interactions**					
Temporo-parietal junction L	918	9.61	−50	−52	10
Temporo-parietal junction R	562	7.64	52	−42	14
Fusiform gyrus R	11	6.53	24	−44	−16
**Non-habitual > unresolved interactions**					
Fusiform gyrus R^∗^	9215	13.00	20	−42	−14
Inferior frontal gyrus, pars opercularis R	493	5.39	48	18	26
Precentral gyrus R	72	4.72	48	2	50

*Clusters with a minimum cluster size of 10 voxels and located within the brain mask are reported. T- or F-values are reported at p_FWE_ < 0.05. Peak voxel coordinates are given in MNI-space. ^∗^Cluster extends to left hemisphere.*

## Discussion

We identified types of non-verbal social interactions in a movie narrative based on a coding scheme derived from the semiotic framework of Peirce’s UCs and segregated neural processes during the observation of such interactions. Three non-verbal interaction types were determined: unresolved (predominant Firstness Category), non-habitual (Secondness), and habitual (Thirdness). These non-verbal interactions together with the verbal ones yielded activations in brain structures involved during naturalistic viewing conditions. The non-verbal interactions yielded lower auditory activation than verbal ones, but significant effects of type emerged: Unresolved events primed auditory cortex, whereas habitual ones suppressed it. Increased activity after non-verbal compared to verbal interactions in the visual cortex and right lateral PFC was due to non-habitual interactions. Further exploration across interaction types revealed that non-habitual interactions yielded higher TPJ activations than unresolved and, most markedly, habitual interactions. The latter recruited the parahippocampal gyrus and lateral occipital complex (LOC) more than unresolved interactions. In summary, Peirce’s cognitive-semiotic categories distinguished three modes for interpreting social interactions. Their underlying cognitive mechanisms led to neural representations of perception and interpretation during the observation of an ongoing film sequence.

### Neural Responses to Social Interactions

Widespread brain regions encompassing visual, auditory, and object-processing areas responded to social interactions shown in a movie. Similar patterns of activation have been observed when contrasting social versus non-social contents depicted in photographs (Deuse et al., 2016) and movie clips (Iacoboni et al., 2004; Wolf et al., 2010; Lahnakoski et al., 2012). In particular, the reported brain structures comprised bilateral fusiform cortex, superior temporal cortex extending into the TPJ, right inferior frontal cortex, and dorsomedial PFC. Such pattern is readily expected from sensory and perceptual processing, but lacks involvement of the language networks (i.e., left IFG “Broca” and pSTG “Wernicke”). Similarly, under naturalistic stimulation conditions in previous studies not explicitly investigating language processing, language network responded weakly only (Malinen et al., 2007; Jääskeläinen et al., 2008; Wilson et al., 2008). The weak recruitment of prefrontal structures in our study (not surpassing the corrected threshold) may be explained by the lack of an explicit task aiming at social cognition (Nummenmaa and Calder, 2009; Nardo et al., 2016). Indeed, frontal-cortical activation was found to be variable during film clips (Hasson, 2004; Kauppi et al., 2017). Taken together, the content-coded social interactions yielded activation patterns in established networks for perceptual processes and social cognition, yielding validity to our event related coding of social interactions during naturalistic stimulation.

### Neuronal Correlates of Interactions Types

The contrast between non-verbal and verbal interactions separated networks for auditory and visual processing. As expected, the bilateral STG responded most strongly to verbal interactions, whereas non-verbal interactions activated visual pathways. The latter finding suggests that during verbal stimulation visual cues are less processed (Wolf et al., 2014). In addition to this clear pattern, the non-verbal, non-auditory interactions yielded a cross-modal modulation in the bilateral STG extending into the MTG. Both the ROI analysis and the comparative mapping across interaction types revealed that STG recruitment was increased for unresolved interactions and was decreased for habitual interactions. Attempts to disambiguate unresolved, socially relevant interactions may prime temporal auditory areas to seek additional auditory cues. In a similar vein, during an audio-visual emotion judgment task, the influence of one modality was greater when the other modality provided ambiguous information (Massaro and Egan, 1996). This effect is thought to be based on cross-modal modulation of basic perception. For instance, magnetoencephalographic studies reported anticipatory pre-activation of auditory cortex by visual motion cues (Hertrich et al., 2007) and modulatory effects of predictability in an audio-visual apparent motion task (Zvyagintsev et al., 2008). The cross-modal priming effect also enhances processing of communication and interaction. A modulation of superior temporal regions supporting auditory processing has been reported for changes in perceived communicative intent (Biau et al., 2016), contextual embedding of speech (Skipper, 2014), stimulus familiarity (Hein et al., 2007), and perceived intentionality of actions (Pelphrey et al., 2004). Taken together, the STG and MTG were sensitive to interpretation processes for communicative actions and interactions. Thus, in the absence of speech, ambiguous visual information (unresolved social interactions, Firstness category) increased neural involvement in processing anticipated speech and behavior.

Non-verbal interactions recruited visual areas and the right lateral PFC more than verbal interactions. This activation was entirely due to responses to non-habitual interactions (Secondness category) and not to the unresolved or habitual ones. It seems that observing non-habitual interactions required enhanced visual processing. Implication of the right lateral PFC regions indicates that the processing focus may be particularly on the contextualization of action and movement patterns. There is evidence that both the right IFG and MFG are involved in the representation of goal-directed aspects of actions (Koski et al., 2002) and in the interpretation of actions in social contexts (Decety and Sommerville, 2003). Indeed, the lateral PFC supports learning and employing rule knowledge relevant to actions (for a review, see Bunge, 2004). More specifically, the right PFC contributes to the creation and testing of rules (Bunge et al., 2003; Donohue et al., 2005), particularly when these need to be integrated into contextual information (Lenartowicz et al., 2010; Waskom et al., 2014). In previous research, the involvement of the lateral PFC during the learning and application of rules has been implicated in both concrete experiential tasks and the formation of abstract behavior-independent rules (Badre et al., 2010; Waskom et al., 2014), and, further, to patterns of appropriate behavior within a given social context (Burgess et al., 2000; Goel et al., 2004; Bolling et al., 2011). In the same vein, the right IFG was related to contextual integration involved in the comprehension of unconventional communicative object configurations (Tylén et al., 2009). Our finding that observing non-habitual interactions recruits right IFG/MFG contributes to this line of research. When encountering a behavior which discords with context-based expectations, new hypotheses of social rules and intentions need to be formulated; this process may recruit right IFG/MFG. This view is in agreement with the conceptualizing of Secondness by Peirce, where the interpreter contextualizes signs and has not yet formed rules (Peirce, 1960).

In non-habitual interactions the observed agent did not act in accordance with socially accepted behavior and, therefore, displayed unexpected behaviors that contrasted with the observer’s predictions (Lee and McCarthy, 2015). This may have led, first, to an allocation of attention toward the agent’s actions (Vossel et al., 2014) as well as toward the reaction of the interacting partner, and, second, to a re-evaluation and remodeling of the agent’s intentions and motivations (Pelphrey et al., 2004; Saxe et al., 2004). When directly contrasting non-habitual with habitual interactions and with unresolved interactions, we detected particularly strong bilateral TPJ activation. A susceptibility of TPJ to other people’s intentions was demonstrated for the right TPJ in response to observing incorrect goal-oriented hand/arm movements (Pelphrey et al., 2004), observing motoric and social errors (Jääskeläinen et al., 2016), and intentional whole body action (Saxe et al., 2004). These findings indicate enhanced recruitment of TPJ during the evaluation and modeling of an observed agent’s intentions.

An alternative explanation for increased activation in visual processing areas, TPJ, and right frontal cortex may reside in the increased saliency of unexpected behaviors. Although expectation (stimulus is expected versus unexpected) and attention (stimulus is relevant versus irrelevant) are dissociable mechanisms in the visual system (Summerfield and Egner, 2009), both may have interacted during the observation of non-habitual interactions. Increased activation in the right TPJ and the IFG has been found for experimentally induced competition between several salient visual events during free viewing of movie clips (Nardo et al., 2016). However, the scenes depicted single or interacting people and thus were social in nature as well. Furthermore, a sharp differentiation between salience-induced attentional processes and mentalizing processes (as part of Theory of Mind) may be inconsequential during social cognition. Both processes can be understood in terms of contextual updating, which is a suggested key function of TPJ (Geng and Vossel, 2013). Therefore, during observation of socially relevant scenes, attentional processes may help evaluate the appropriateness of the observed actions and model possible motives and reactions of the interacting partner.

### Habitual Non-verbal Interactions

The contrasts between verbal and non-verbal interactions have elucidated the distinct contributions of unresolved and non-habitual interactions. Further, specific effects for habitual interactions (Thirdness category) emerged in the direct contrasts between non-verbal interaction types. The bilateral LOC and the left parahippocampal gyrus were activated more in response to habitual than to unresolved interactions. Habitual – and thus rule-conforming – actions are well-learned and regularly encountered in everyday life. Therefore, visual scene analysis and matching input to memory representations may dominate the interpretation process. The LOC is traditionally associated with processing and recognizing faces, body parts, and goal-directed movements as well as general motion patterns (for a review, see Lingnau and Downing, 2015). The parahippocampal gyrus contributes to associative memory and visuo-spatial processing (for a review, see Aminoff et al., 2013). Therefore, the LOC-parahippocampal network may represent real-world scenes complementing the perception of photographs (Park et al., 2011) and movie clips (Lahnakoski et al., 2014). Interpreting interactions as habitual and conforming to social rules seems to be associated with processing in object-recognition and memory structures.

Habitual interactions are not only well-learned but also conform to schematic behavioral patterns and social rules. The LOC-parahippocampal network may additionally contribute to the extraction, recognition, and contextualization of behavioral patterns. Involvement of the LOC has been reported for contextual guidance during visual search in complex scenes (Lin and Scott, 2011), the creation of category-level templates for recognizing humans and objects (van Koningsbruggen et al., 2013), and the abstraction of actions from agents (Kable and Chatterjee, 2006). Similarly, increased activation in the parahippocampal gyrus has been reported both for goal-oriented actions (Lahnakoski et al., 2012) and for more abstract functions of scene analysis such as scene categories (Peelen and Kastner, 2014), locational concepts (Huth et al., 2016), and perceptual schema representations (Bar, 2009). These processes may contribute to the understanding of action schemas, which are, on a mechanistic level, the basis of habitual social interactions. Thus, our results indicate that during the observation of habitual interactions, the parahippocampal gyrus and the LOC interact to evaluate the observed action patterns.

### Universal Categories and Neural Activation Patterns

The interpretation of social interactions requires synergy of various cognitive functions raging from multimodal perception and social attention to language comprehension and mentalizing (Ochsner and Lieberman, 2001; Adolphs, 2009; Hari and Kujala, 2009). As novel means to a comprehensive typology, Peirce’s semiotic framework of UCs was utilized to inform three types of social interactions. These interaction types were presented in a movie context and yielded distinct neural patterns. The Firstness category is predominant in unresolved social interactions, which recruited bilateral STG. Conceivably, in ambiguous social situations, attention was directed toward the auditory modality (e.g., Massaro and Egan, 1996), as is predicted by the “principle of inverse effectiveness” (Stein and Meredith, 1993). The perception of a stimulus can be altered by a cue from another modality (Klasen et al., 2012a); in particular, audiovisual stimuli of lower intensity yield larger recognition benefit that those with higher intensity (Diederich and Colonius, 2004; Rach et al., 2011). During unresolved interactions, when information density of the visual modality was reduced, auditory processing increased. Spoken language is highly codified and thus effectively resolves a situation.

Social interactions of the Secondness category (non-habitual) required the highest processing demand in visuo-spatial, mentalizing, and contextual rule-learning areas (IFG). This pattern suggests increased attention toward the unexpected behavior and a re-evaluation of the observed agent’s intentions within the situational context (Lee and McCarthy, 2015). This mismatch between prior expectations and reality triggers a cognitive prediction error, which has been associated particularly with dorsolateral PFC (Fletcher et al., 2001; Turner, 2004). Prediction error processing elicits attentional orienting and underlies mentalizing and contextual learning (den Ouden et al., 2012).

The observation of habitual interactions (Thirdness category) recruited brain regions supporting conceptual processing and associative memory retrieval. Habitual behaviors may be encoded as learned action patterns and social schemas (Bolling et al., 2011), Social schemas are memory representations of typical contexts (Spalding et al., 2015), which guide and facilitate the processing of social information (Augoustinos and Innes, 1990) and their reconstruction from memory (Bartlett, 1932); the latter involves the ventromedial PFC regions (Spalding et al., 2015) rather than lateral PFC.

Taken together, characterizing non-verbal social interactions with Peirce’s cognitive-semiotic categories enabled the holistic neurosemiotic investigation of complex social cognition. Such a semiotic approach offers a novel means to investigate the neural representation of communication in naturalistic stimuli.

Building on Peirce’s semiotic theory, various theoretical frameworks highlight specific aspects of communicative signs. For instance, the UCs have been instrumental to empirical analyses of manual gestures (McNeill, 1992, 2005; Fricke, 2007; Mittelberg, 2008, 2013, 2018; Mittelberg and Waugh, 2014). McNeill’s Peirce-inspired differentiation of gestures into iconic gestures (icon, Firstness category), deictic gestures (index, Secondness category), and emblems (symbol, Thirdness category; McNeill, 1992, 2005) has become a prominent strand within gesture research. Neuroimaging studies on gesture perception and comprehension revealed that manual gestures in general recruited the language systems (IFG and posterior superior temporal sulcus) and the action-movement systems (inferior parietal and premotor cortex; Andric and Small, 2012) whereas iconic gestures, representing salient visual features of an object referred to in speech, activated a fronto- posterior temporal network (Özyürek, 2014). Furthermore, emblems, being symbolic and highly conventionalized signs, recruited the language networks (Bernardis and Gentilucci, 2006; Xu et al., 2009).

Symbolic and conventionalized meaning corresponds to Thirdness in Peirce’s UCs (Peirce, 1960; Potter, 1967; Bateman et al., 2017) and is considered in neurocognitive investigations not only for gesture comprehension (Villarreal et al., 2012; Andric et al., 2013) but also for the perception of communicative signs in a broader sense (Donohue et al., 2005; Sato et al., 2009; Pulvermüller, 2013). For instance, pictures of objects activated the language network (like words) if they were perceived as symbolic (Tylén et al., 2009) or when they conveyed abstract social meaning (Tylén et al., 2016). Furthermore, Peirce’s UCs have inspired theories regarding the emergence of social conventions and symbolic communication during language evolution (Deacon, 1997) and during child development (Daddesio, 1994). These examples emphasize the applicability of Peirce’s semiotics for the investigation of communicative signs and behaviors with respect to various signal properties. However, since empirical studies are still scarce and the operationalizations vary across studies, the picture is yet incoherent and impedes generalizations (Fusaroli and Paolucci, 2011; Zlatev, 2012). The application of UCs has a high potential with regards to investigating communication processes and should be further explored in the context of social learning or social impairments such as seen in patients with schizophrenia or autism spectrum disorders.

### Methodological Considerations and Limitations

In order to relate functional responses to the social interaction types we conducted a GLM analysis and corrected the resulting brain maps with voxelwise FWE correction (*p*_FWE_ < 0.05). To minimize spurious activation voxels, only cluster with size according to a cluster-wise FWE correction are reported. Furthermore, localizations were confirmed with a Neurosynth-based a priori mask, with one exception. The cluster in right MFG for non-verbal interactions over verbal interaction failed to overlap with the mask and, thus, may constitute a false positive result and needs to be confirmed in follow up studies.

The unequal distribution of events and event-durations introduces differences in the amount of observations. This heterogeneity may cause a violation of the heteroscedasticity assumption for analyses of variance. Therefore, the model estimation was done with assuming unequal-variance as provided by SPM. Thus we minimized the statistical bias due to the violation of the heteroscedasticity assumption of the regressors. The imbalance of event numbers and durations constitutes a limitation of the analysis, but also reflects the nature of naturalistic stimulation. Naturalistic stimuli such as movie clips are inherently complex and contain a multitude of diverse, dynamic, and interacting information. Although natural viewing conditions offer superior ecological validity compared to more traditional experimental paradigms, the stimulus conditions are less well controlled (Hasson, 2004). The number of events and the total duration vary across the coded interaction types. With our content-coding based GLM approach, we aimed to model the neural responses to social interaction types by generalizing across movie scenes and, therefore, by and large independently of other movie contents. However, since the presentation of events is not controlled or randomized, the stimuli may differ in complexity and may coincide with other influencing factors or physical characteristics. Nevertheless, the congruent neural patterns to other studies investigating social interactions lend credibility to this pseudo-experimental design, as shown in previous studies employing similar content-coding methodology (e.g., Mathiak and Weber, 2006).

The movie “Lola rennt” comprised a large variety of social situations and scenes as well as different cinematographic elements and thus may be considered a comprehensive naturalistic stimulus; nevertheless, the activation patterns may be stimulus-specific and may not generalize to other movie-excerpts. Until now, movie excerpts were mainly analyzed with data driven methods such as ISC and independent-component analysis (ICA), instead of a model-based approach. Therefore, additional studies using model-based analyses need to determine stimulus-independence and generalizability of the here presented activation patterns.

Peirce’s pragmatic approach to communication processes makes his theory well suited for systematic analyses (Mittelberg, 2008, 2018; Bateman et al., 2017). Our content-coding based design is a theory-driven approach to iteratively obtain a meaningful typology of non-verbal social interactions (Weber et al., 2009). The coding results show a good reliability. Validity is, on the one hand, established by the theoretical foundation and, on the other hand, confirmed by the meaningful neural contributions (Pajula et al., 2012). The approach based on semiotic categories facilitates neurocognitive analysis; however, additional non-categorical, continuous measures may reflect interaction-related features in more detail. Further refinements of the operationalization can target not only the validity of the coding but may employ the UCs to also describe other processes that underlie the perception and comprehension of various kinds of signs.

## Conclusion

We operationalized Peirce’s semiotic typology to describe basic social cognitive categories of non-verbal interactions in a movie narrative. Functional imaging revealed specific and meaningful responses in the brain to the observed events. Firstness: During the observation of unresolved interactions, the ambiguous visual information enhanced neural involvement in bilateral STG even in the absence of speech – conceivably as a cognitive mechanism to attend to additional resolving cues in another modality. Secondness: In response to non-habitual interactions that contrasted contextual expectations, the visual and prefrontal cortices as well as the TPJ supported the interpretation of intentions and the re-evaluation of social rules. Thirdness: The interpretation of habitual interactions recruited neural correlates for object recognition and associative memory. Semiotic approaches may help to elucidate mechanisms of social communication beyond confined linguistic theories.

## Author Contributions

DW have designed, tested and applied the content coding scheme, analyzed and interpreted the data, and wrote the manuscript. IM have applied the semiotic theory on content coding scheme and edited the manuscript. L-MR have applied the semiotic theory on content coding scheme and edited the manuscript. SB have planned the study and collected the data. MZ have implemented the fMRI measurement details and assisted in data collection. AH have designed, tested, and applied the content coding scheme. MK have assisted in designing the content coding scheme and edited the manuscript. FC have counselled and assisted in data analysis. KM have planned the study, assisted in data analysis and interpretation, and edited the manuscript.

## Conflict of Interest Statement

The authors declare that the research was conducted in the absence of any commercial or financial relationships that could be construed as a potential conflict of interest.

## Footnotes

1 https://neurovault.org/collections/4075/

2 www.neurosynth.org
