# Supplementary material for: Interpretation of Social Interactions: Functional Imaging of Cognitive-Semiotic Categories During Naturalistic Viewing
Source: Front Hum Neurosci. 2018 Aug 14;12:296. doi: 10.3389/fnhum.2018.00296 (PMC6102316; doi:10.3389/fnhum.2018.00296)
Supplement: Supplementary file 1 [file Data_Sheet_1.docx]

**Figure S1:**


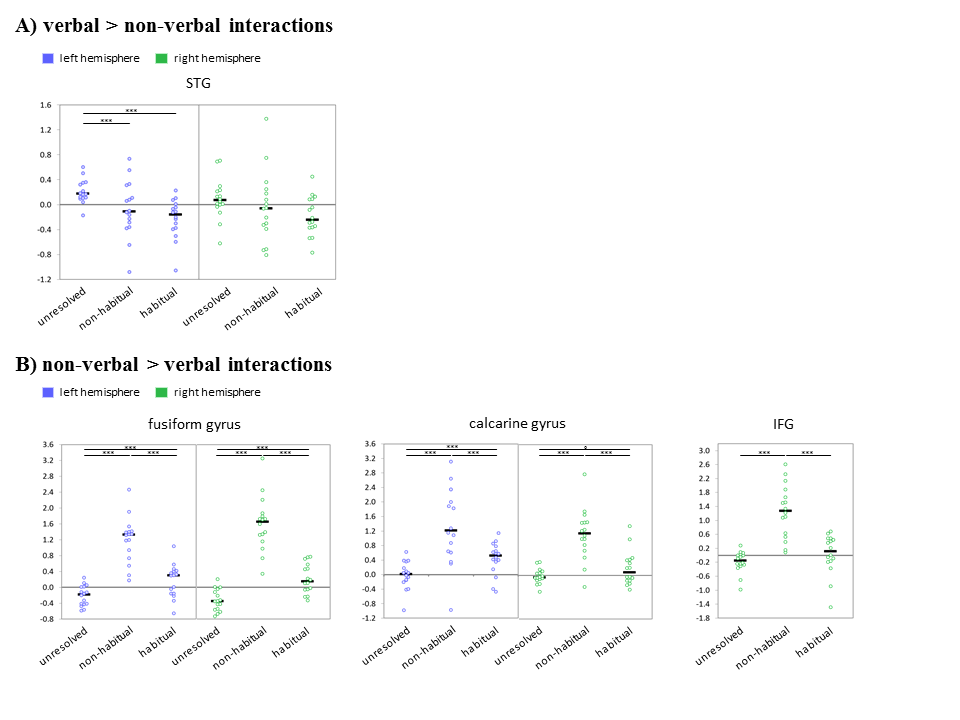


**Figure S1:** The contrasts between verbal (‘verbal > non-verbal’) and non-verbal (‘non-verbal > verbal’) interactions separated the networks for visual-based and sound-based information processing (compare Fig. 3). The obtained brain maps were masked with small-volume correction informed by the merged neurosynth images ‘verbal’ and ‘social interaction’. The resulting peak voxels confirmed the specific contributions for (A) verbal interactions and (B) the non-verbal interaction types (‘unresolved’, ‘non-habitual’, and ‘habitual’). The cluster previously obtained in right middle frontal gyrus was not covered by the neurosynth-based mask. Abbreviations: LH: left hemisphere; RH: right hemisphere; IFG: inferior frontal gyrus; MFG: middle frontal gyrus; STG: superior temporal gyrus; *: p < .05; **: p < .01; ***: p < .001; °: p < .1 (trend); z- coordinates are indicated beneath each slice.

**Figure S2:**


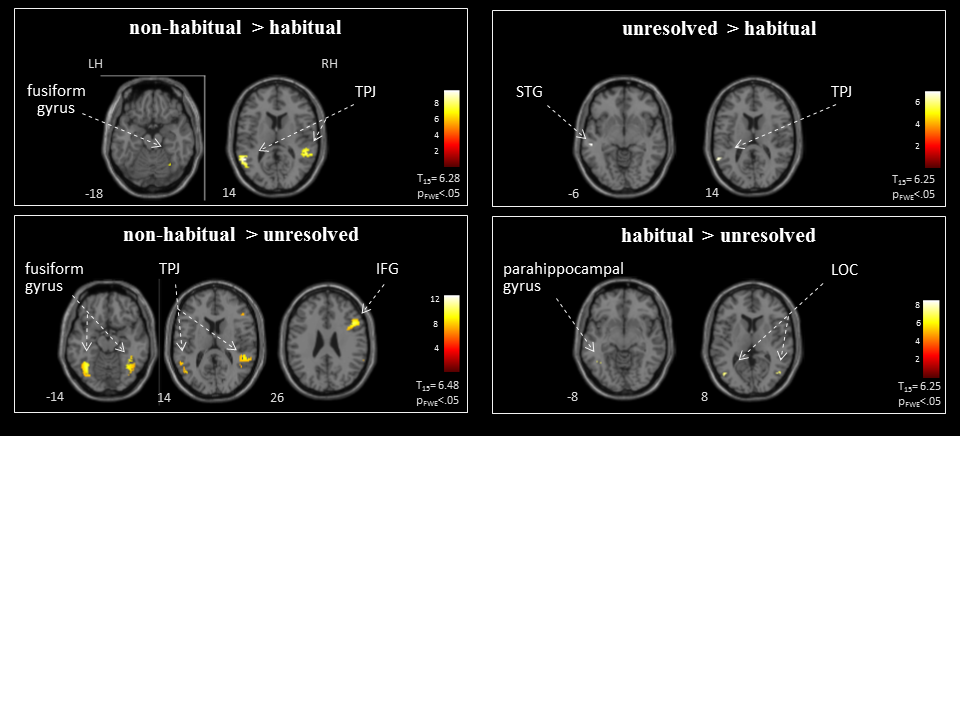


**Figure S2:** Neural networks for the processing of non-verbal interactions as corrected with the neurosynth mask.

Post-hoc t-maps determined specific effects of the non-verbal interactions types in four directed contrasts (compare Fig. 3). Each contrasts was small-volume corrected with the neurosynth-based brain mask. For visualization the contrasts were masked with the neurosynth-based mask. The reported clusters for each contrasts are located within the brain mask – at least in part – thus validating the findings with a meta-analytical approach. Abbreviations: LH: left hemisphere, RH: right hemisphere; IFG: inferior frontal gyrus; LOC: lateral occipital complex; pSTG: posterior superior temporal gyrus, MFG: middle frontal gyrus; MOG: middle occipital gyrus; SOG: superior occipital gyrus; TPJ: temporo-parietal junction; z-coordinates are indicated beneath each slice.

| **Table S1:** Cluster table for comparison between non-verbal interaction types corrected with neurosynth-based mask. | | | | | |
| --- | --- | --- | --- | --- | --- |
| peak voxel location | cluster size [voxel] | peak t-value | peak voxel | | |
|  |  |  | x | y | z |
| **unresolved > habitual interactions** |  |  |  |  |  |
| middle temporal gyrus L | 30 | 7.00 | -56 | -26 | -6 |
| temporo-parietal junction L | 19 | 6.71 | -58 | -52 | 14 |
|  |  |  |  |  |  |
| **habitual > unresolved interactions** |  |  |  |  |  |
| inferior temporal gyrus L | 1 | 6.58 | -44 | -54 | -8 |
| middle occipital gyrus L | 27 | 8.49 | -44 | -74 | 12 |
| middle occipital gyrus L | 2 | 6.96 | -32 | -74 | 24 |
| middle occipital gyrus L | 1 | 8.01 | -34 | -78 | 20 |
| middle occipital gyrus L | 1 | 6.48 | -30 | -80 | 20 |
| middle occipital gyrus L | 1 | 6.44 | -38 | -64 | 0 |
| middle occipital gyrus L | 1 | 6.26 | -38 | -64 | -4 |
| middle occipital gyrus R | 27 | 7.37 | 46 | -72 | 6 |
| parahippocampal gyrus L | 1 | 6.51 | -38 | -56 | -8 |
|  |  |  |  |  |  |
| **non-habitual > habitual interactions** |  |  |  |  |  |
| fusiform gyrus (extending into cerebellum) R | 18 | 6.96 | 24 | -64 | -20 |
| middle temporal gyrus R | 3 | 6.51 | 50 | -30 | -6 |
| supramarginal gyrus R | 3 | 6.65 | 58 | -46 | 24 |
| supramarginal gyrus R | 1 | 6.48 | 64 | -46 | 26 |
| temporo-parietal junction L | 410 | 9.60 | -52 | -52 | 12 |
| temporo-parietal junction R | 180 | 7.64 | 52 | -42 | 14 |
|  |  |  |  |  |  |
| **non-habitual > unresolved interactions** |  |  |  |  |  |
| calcarine gyrus R | 10 | 7.28 | 10 | -80 | 6 |
| calcarine gyrus R | 1 | 8.14 | 14 | -88 | 2 |
| fusiform gyrus L | 888 | 9.76 | -34 | -62 | -20 |
| fusiform gyrus R | 472 | 12.48 | 38 | -58 | -24 |
| fusiform gyrus R | 1 | 9.21 | 28 | -62 | -12 |
| fusiform gyrus R | 1 | 6.90 | 38 | -74 | -14 |
| **Table S1** cont. | | | | | |
| peak voxel location | cluster size [voxel] | peak t-value | peak voxel | | |
|  |  |  | x | y | z |
| inferior frontal gyrus, pars opercularis R | 315 | 9.75 | 48 | 18 | 26 |
| inferior frontal gyrus, pars triangularis R | 7 | 6.68 | 48 | 30 | 14 |
| lingual gyrus R | 29 | 9.11 | 14 | -84 | -8 |
| lingual gyrus R | 2 | 8.11 | 16 | -82 | 0 |
| middle occipital gyrus L | 4 | 7.35 | -28 | -82 | 16 |
| middle occipital gyrus L | 1 | 6.50 | -44 | -74 | -2 |
| middle temporal gyrus R | 1 | 6.50 | 46 | -62 | 16 |
| precentral gyrus R | 14 | 7.22 | 46 | 2 | 44 |
| supramarginal gyrus R | 1 | 6.69 | 64 | -46 | 26 |
| temporo-parietal junction R | 6 | 6.91 | 46 | -58 | 12 |
|  |  |  |  |  |  |
| Clusters located within the brain mask are reported. T- or F-values are reported at p_FWE_ < .05; peak voxel coordinates are given in MNI-space. | | | | | |
